# Supplementary material for: Histone methyltransferase DOT1L coordinates AR and MYC stability in prostate cancer
Source: Nat Commun. 2020 Aug 19;11:4153. doi: 10.1038/s41467-020-18013-7 (PMC7438336; doi:10.1038/s41467-020-18013-7)
Supplement: Supplementary file 1 — Supplementary Information [file 41467_2020_18013_MOESM1_ESM.pdf]

Histone Methyltransferase DOT1L Coordinates AR and MYC Stability in Prostate Cancer

Vatapalli et al.

Supplementary Fig 1.

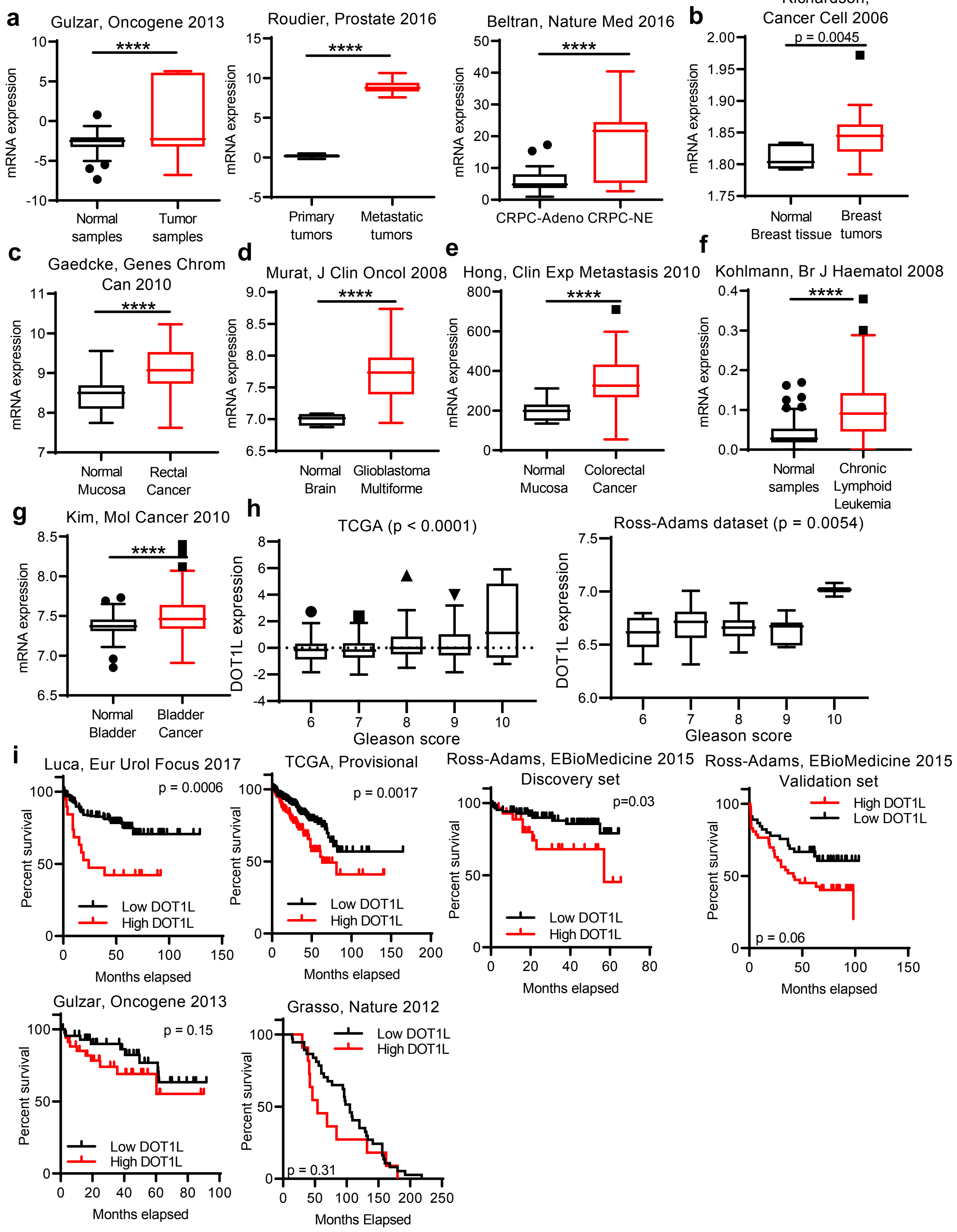

Supplementary Fig 1: (a) Comparison of *DOT1L* expression in three cohorts of prostate cancer patients. Data for this analysis was used from Gulzar Prostate cancer dataset [normal (n=66), prostate cancers (n=83) and Beltran dataset [CRPC adenocarcinomas (n=15), the Roudier Prostate cancer dataset [primary cancers (n=11), metastatic cancers (n=45)], CRPC-Neuroendocrine cancers (n=31)]. (b-g) Expression of *DOT1L* in multiple cancer types. Data was used from (b) Richardson Breast cancer dataset [Normal (n=7), Cancer (n=40)], (c) GaedckeRectal cancer dataset [Normal (n=65), Cancer (n=65)], (d) Murat Glioblastoma dataset [Normal (n=4), Cancer (n=80)], (e) Hong Colorectal cancer dataset [Normal (n=12), Cancer (n=70)], (f) Kohlmann CLL dataset [Normal (n=73), Cancer (n=448)], (g) Kim Bladder cancer [Normal (n=68), Cancer (n=188)]. (h) Comparison of *DOT1L* expression with Gleason score in two cohorts of prostate cancer patients. Data was used from TCGA dataset and Ross-Adams discovery set. P-value determined by ANOVA. (i) Disease free Survival analysis of six independent cohorts of prostate cancer patients divided by expression of *DOT1L*. Data for this analysis was used from the Luca CancerMap prostate cancer dataset [Cut-off at 90th percentile, high *DOT1L* (n=19), low *DOT1L* (n=214)], TCGA prostate cancer dataset [Cut-off at 90th percentile, high *DOT1L* (n=33), low *DOT1L* (n=463)], Ross-Adams Discovery dataset [Cut-off at 75th percentile, high *DOT1L* (n=24), low *DOT1L* (n=84)], Ross-Adams Validation dataset [Cutoff at 50th percentile High(n=92), Low (n=92), p=0.0632] and Gulzar dataset [Cutoff at 50th percentile, High (n=83), Low (n=83), p=0.1535]. Overall survival data was used from Grasso dataset [High (n=48), Low (n=48), p=0.3118]. Statistical tests: P value determined by two-sided Welsh's t-test (a-g) and Log-rank test (h-i). For box plots, minima and maxima values are shown (a-g). ****p<0.0001.

Supplementary Fig 2

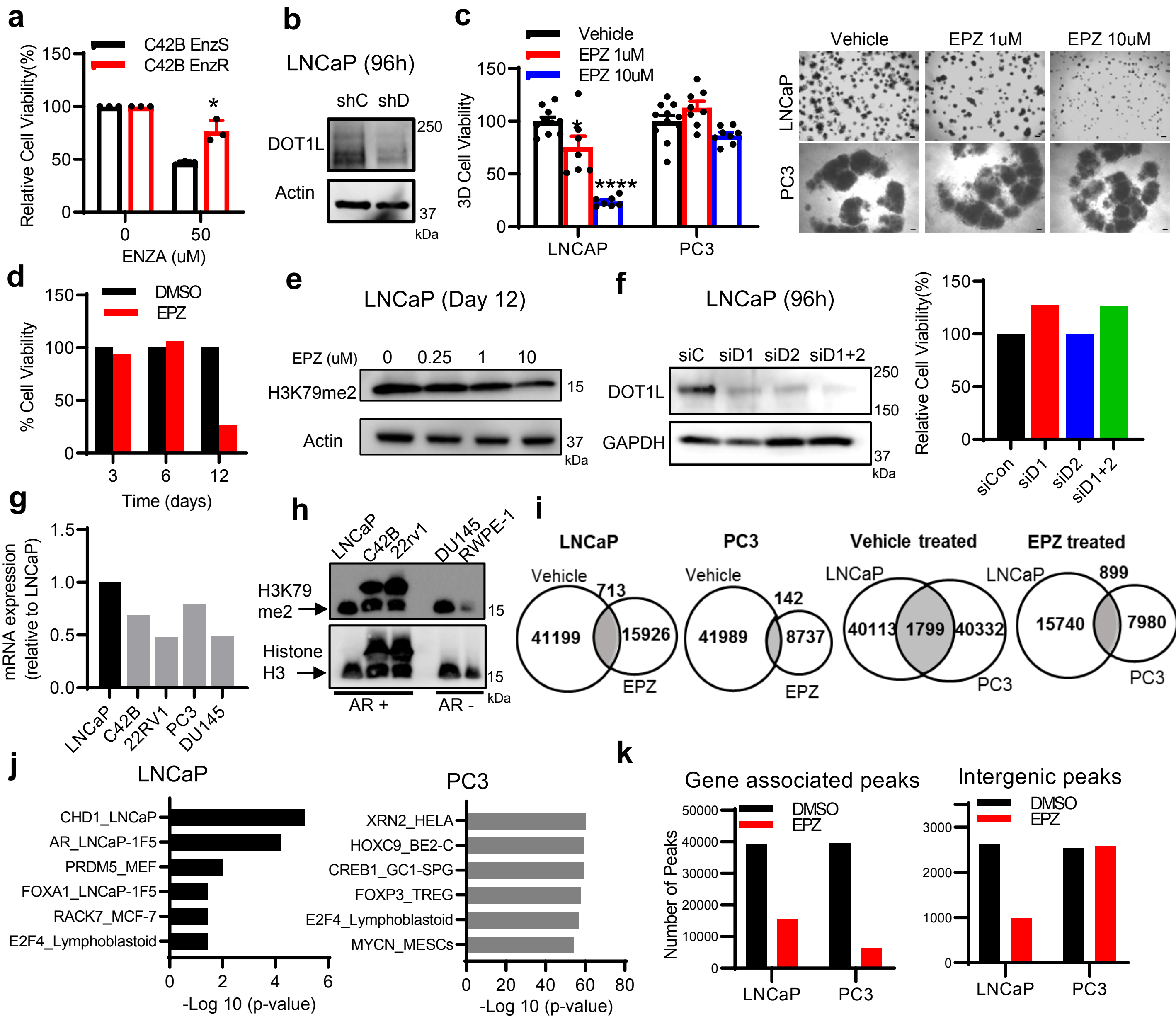

Supplementary Fig 2: (a) Cell viability of C42B-EnzS and C42B EnzR cells treated with 50uM Enza for 2 days. (b) DOT1L western blot in LNCaP cells transduced with Luciferase targeting shRNA or DOT1L targeting shRNA (4 days). (c) 3D Cell viability assay in LNCaP and PC3 organoids after 12 days of EPZ treatment. Representative images shown (right). Scale bars indicate 100uM. (d) Cell viability assays performed after 3,6 and 12 days of treatment with Vehicle or 10uM EPZ in C42B cells. (n=1) (e) H3K79me2 western blot in LNCaP cells after EPZ treatment after 12 days (f) (left) DOT1L western blot (4 days) and (right) Cell viability assays (12 days) in LNCaP cells transfected with Control or 2 single DOT1L targeting siRNA and 1 pool of DOT1L targeting siRNA (n=1) (g) Baseline DOT1L mRNA expression in a panel of Prostate cancer cells – LNCaP, C42B, 22rv1, PC3, DU145. (h) Baseline H3K79me2 western blot in a panel of Prostate cancer cells – LNCaP, C42B, 22rv1, DU145, RWPE-1. (i) Venn diagrams comparing H3K79me2 enriched peaks in LNCaP and PC3 after treatment with Vehicle or 1uM EPZ for 8 days. (j) ChIP Enrichment Analysis (ChEA) of unique H3K79me2 enriched genes from LNCAP and PC3 identified using Enrichr web tool. (k) Comparison of the number of gene associated and intergenic peaks in LNCaP and PC3 cells after treatment with 1uM EPZ for 8 days. Statistical tests: P-values determined by two-tailed student's t-test (a,c). n=3 (a-b,e), n=8 (c), n = 1 (g-h) independent experiments. Error bars represent s.e.m. *p<0.05, ** p<0.01, *** p<0.001, ****p<0.0001

Supplementary Fig 3

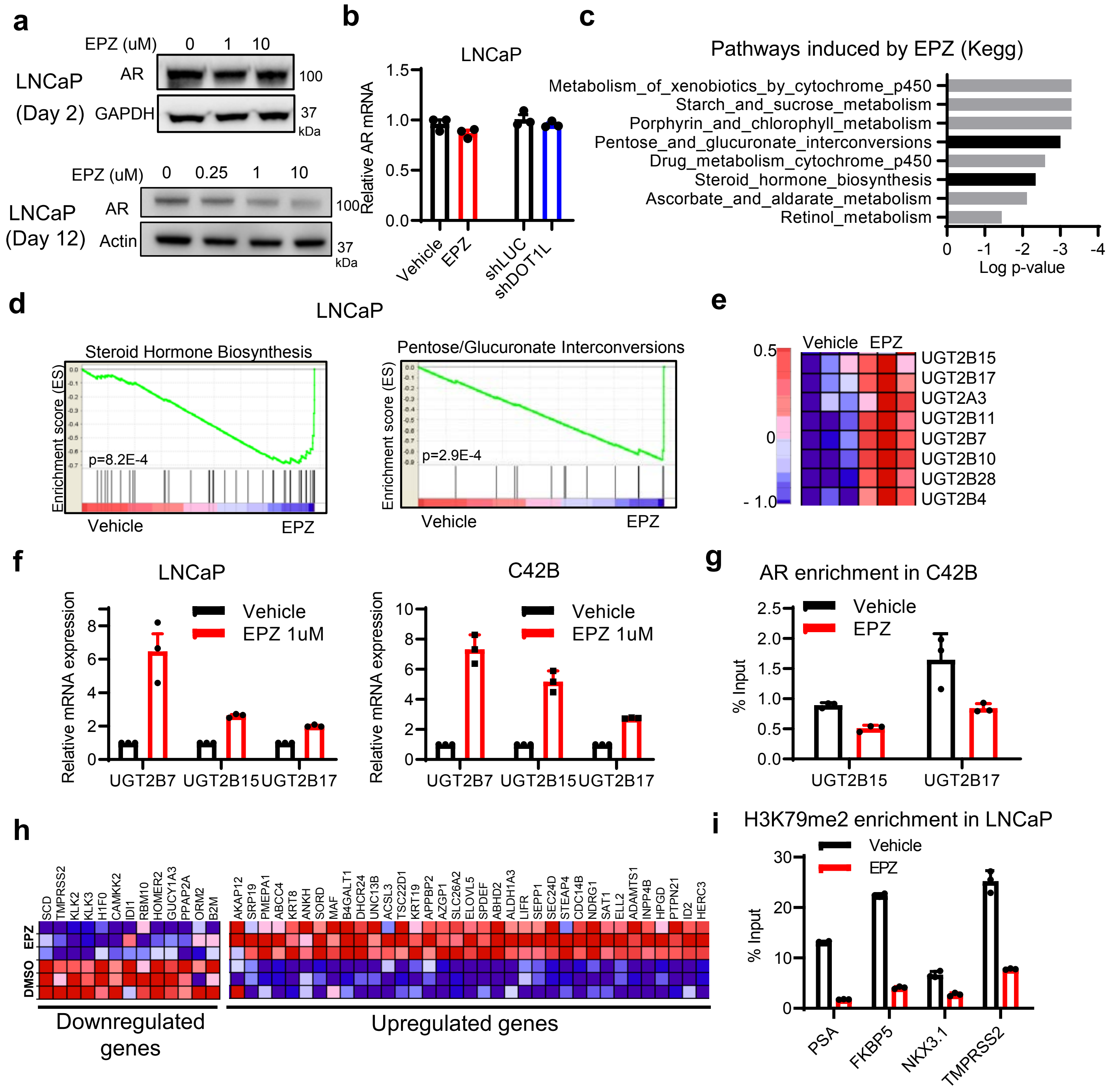

Supplementary Fig 3: (a) Western blot analysis of AR in LNCaP cells treated with Vehicle or indicated doses of EPZ for 2 days and 12 days. (b) *AR* mRNA levels assessed by qPCR in LNCaP cells after EPZ treatment (1uM) and DOT1L knockdown using shRNA after 8 days (c) Top 8 Kegg pathways induced by EPZ identified by GSEA analysis in LNCaP cells after treatment with Vehicle or 1uM EPZ. Bars in black indicate gene-sets of interest. (d) GSEA plot of Steroid_Hormone_Biosynthesis geneset (left) and Pentose_Glucuronate_Interconversions geneset (right) identified by GSEA analysis in LNCaP cells after treatment with Vehicle or 1uM EPZ for 8 days. (e) Heat map of expression of UGT2B family of genes from leading edge of Pentose_Glucuronate _Interconversions gene set. (f) mRNA expression of *UGT2B7, 15*, and *17* after 1uM EPZ treatment in LNCaP and C42B cells for 8 days. (g) AR enrichment at UGT2B gene promoters after Vehicle/EPZ 1uM treatment for 8 days in C42B cells evaluated by ChIP-qPCR. (h) Heatmap of differentially expressed genes from Nelson_Response_To_Androgen dataset identified by GSEA analysis in LNCaP cells treated with Vehicle or 1uM EPZ. (i) H3K79me2 enrichment at AR target genes in LNCaP cells treated with Vehicle or 1uM EPZ for 8 days measured by ChIP-qPCR. Statistical tests: P-values determined by two-tailed student's t-test (b,f-g,i). n=3 (a-b, f-g,i) independent experiments. Error bars represent s.e.m. For GSEA analysis, adjustments were made for multiple comparisons. (c-d) FDR < 25%. *p<0.05, ** p<0.01, *** p<0.001, ****p<0.0001

Supplementary Fig 4

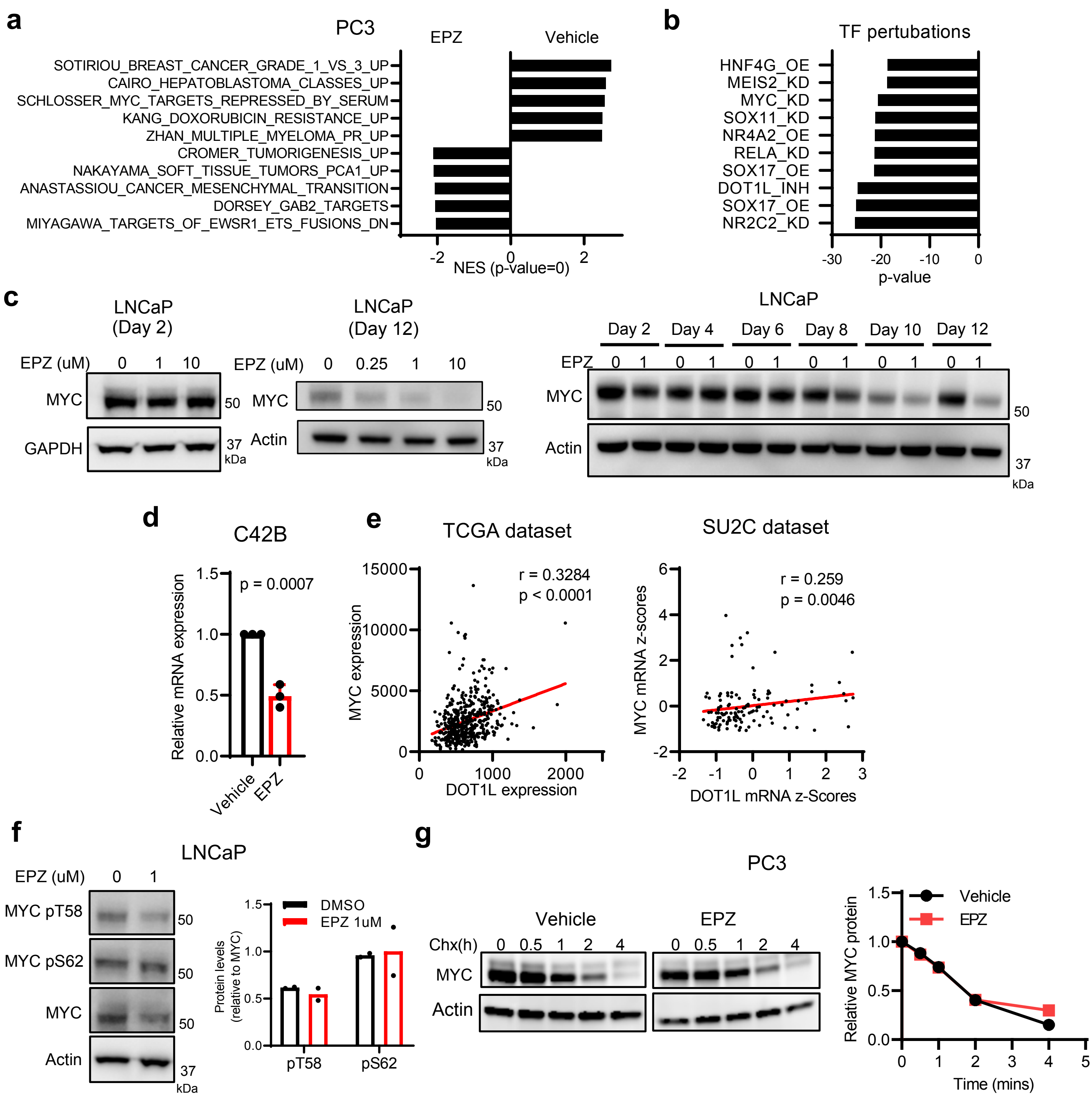

Supplementary Figure 4: (a) Top 5 genesets enriched in PC3 Vehicle and EPZ treated cells identified by GSEA analysis (p value=0). (b) Analysis of the leading edge genes from Schlosser_MYC_Targets_Repressed_By_Serum identifying gene signatures from single TF perturbations using Enrichr web tool. (c) Western blot analysis of MYC in LNCaP cells treated with Vehicle or EPZ at different time points. (d) mRNA levels of *MYC* in C42B cells treated with 1uM EPZ and Vehicle for 8 days. (e) Correlations between gene expression of DOT1L & MYC using data from the SU2C dataset (n=118) and TCGA dataset (n=498). P-values were analyzed using Spearman's rank correlation. (f) Western blot analysis of MYC pT58 and pS62 in LNCaP cells treated with Vehicle or EPZ for 8 days followed by quantitation of protein levels. (g) Western blot analysis and quantitation of MYC protein after treatment with 50 ug/ml Cycloheximide in PC3 cells treated with vehicle or 1uM EPZ for 8 days. Statistical tests: P-values determined by two-tailed student's t-test (d). n = 3 (c-d), n =2 (f), n = 1 (g) independent experiments. Error bars represent s.e.m. For GSEA analysis, adjustments were made for multiple comparisons. (a-b) FDR < 25%. *p<0.05, ** p<0.01, *** p<0.001, ****p<0.0001

Supplementary Fig 5

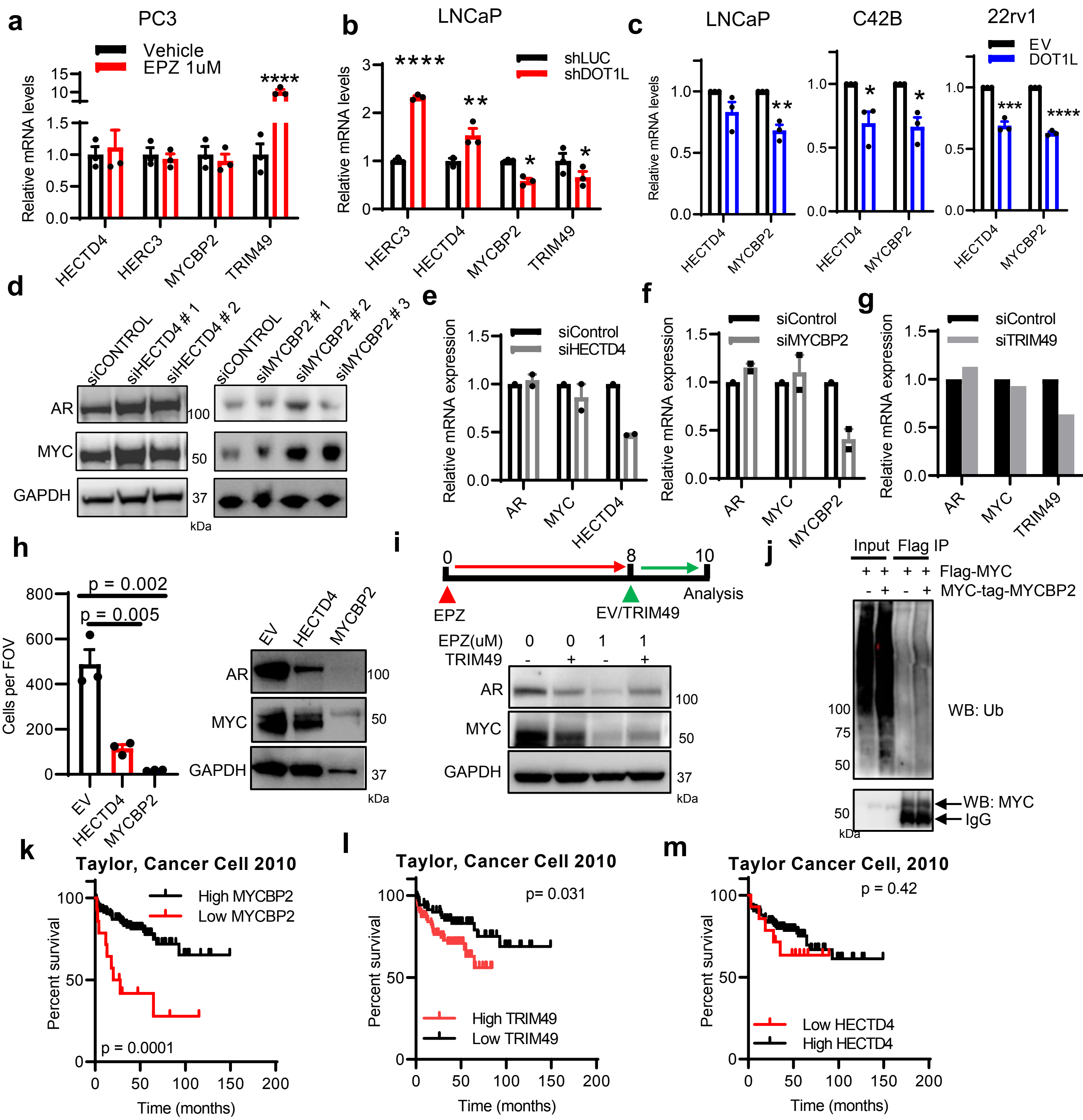

Supplementary Figure 5: (a) mRNA expression of the 4 candidate E3 Ubiquitin ligases in PC3 cells after treatment with 1uM EPZ. (b) mRNA expression of the 4 candidate E3 Ubiquitin ligases in LNCaP cells transduced with shControl or shDOT1L lentivirus. (c) *HECTD4* and *MYCBP2* expression in LNCaP, C42B and 22rv1 cells after transduction with EV or DOT1L expressing constructs. (d) AR and MYC western blot in LNCaP cells transfected with Control or HECTD4/MYCBP2 targeting siRNAs after 2 days. *AR* and *MYC* mRNA expression in LNCaP cells transfected with Control or (e) HECTD4 targeting siRNA, (f) MYCBP2 targeting siRNA, (g) TRIM49 targeting siRNA for 2 days. (h) (left) Average cells per field of view (FOV) in LNCaP cells transfected with HECTD4 and MYCBP2 constructs for 48h (right) AR and MYC protein levels in LNCaP cells transfected with EV, HECTD4, MYCBP2 constructs. (i) AR and MYC protein levels in LNCaP cells treated with Vehicle or EPZ for 8 days followed by transfection of EV or TRIM49 construct for 2 days. (j) Flag-MYC pulldown followed by Ubiquitin western analysis in 293T cells transfected with both Flag-MYC and MYCBP2 constructs. (k-m) Disease free Survival analysis of prostate cancer patients divided by expression of *MYCBP2*, *TRIM49* and *HECTD4*. Data was used from the MSKCC dataset (k) [Cut-off at 10th percentile, high *MYCBP2* (n=126), low *MYCBP2* (n=14)], (l) [Cut-off at median, high *TRIM49* (n=70), low *TRIM49* (n=70)] (m) [Cut-off at 10th percentile, high *HECTD4* (n=126), low *HECTD4* (n=14)]. Statistical tests: P value determined by two tailed t-test (a-c,h), Log-rank test (k-m). n = 3 (a-c,h), n= 2 (e-f,i-j) independent experiments. Error bars represent s.e.m *p<0.05, ** p<0.01, *** p<0.001, ****p<0.0001

Supplementary Fig 6.

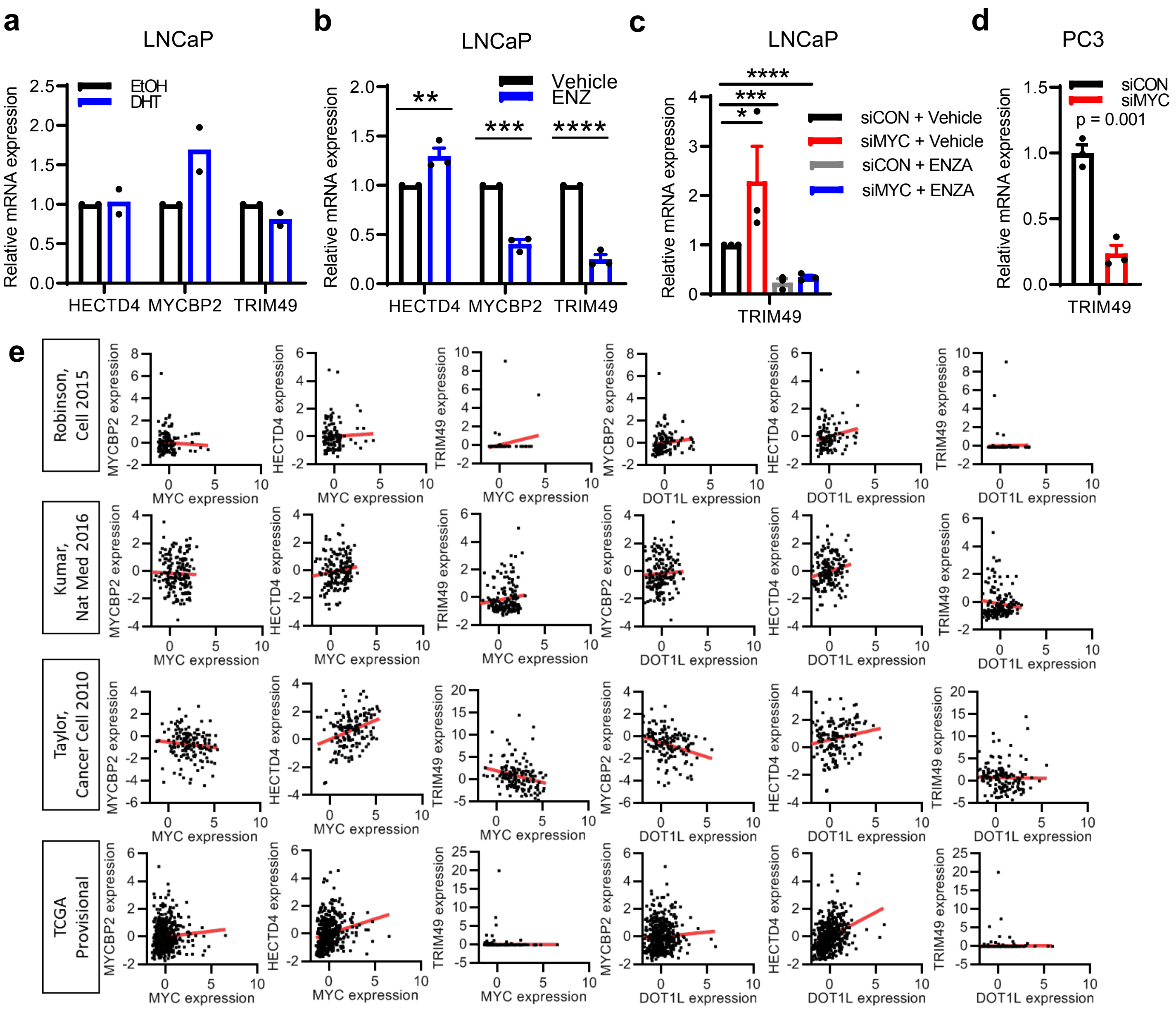

Supplementary Figure 6: (a) mRNA expression of *HECTD4, MYCBP2* and *TRIM49* in LNCaP treated with Control or 10nM DHT for 24 hours in Charcoal stripped media (b) mRNA expression of *HECTD4, MYCBP2* and *TRIM49* in LNCaP treated with Control or 20uM ENZA for 24 hours. (c) mRNA expression of *TRIM49* in LNCaP cells transfected with Control or MYC targeting siRNA (2 days) followed by treatment with Vehicle or 20uM ENZA (2 days). (d) mRNA expression of *TRIM49* in PC3 cells transfected with Control or MYC targeting siRNA for 2 days. (e) Correlation between MYC or DOT1L expression and expression of the three E3 ligases *MYCBP2, HECTD4, TRIM49*. Statistical tests: P value determined by two –tailed Welsh's t-test (b-d). n = 2 (a), n=3 (b-d) independent experiments Error bars represent s.e.m *p< 0.05, ** p<0.01, *** p<0.001, ****p<0.0001

Supplementary Fig 7.

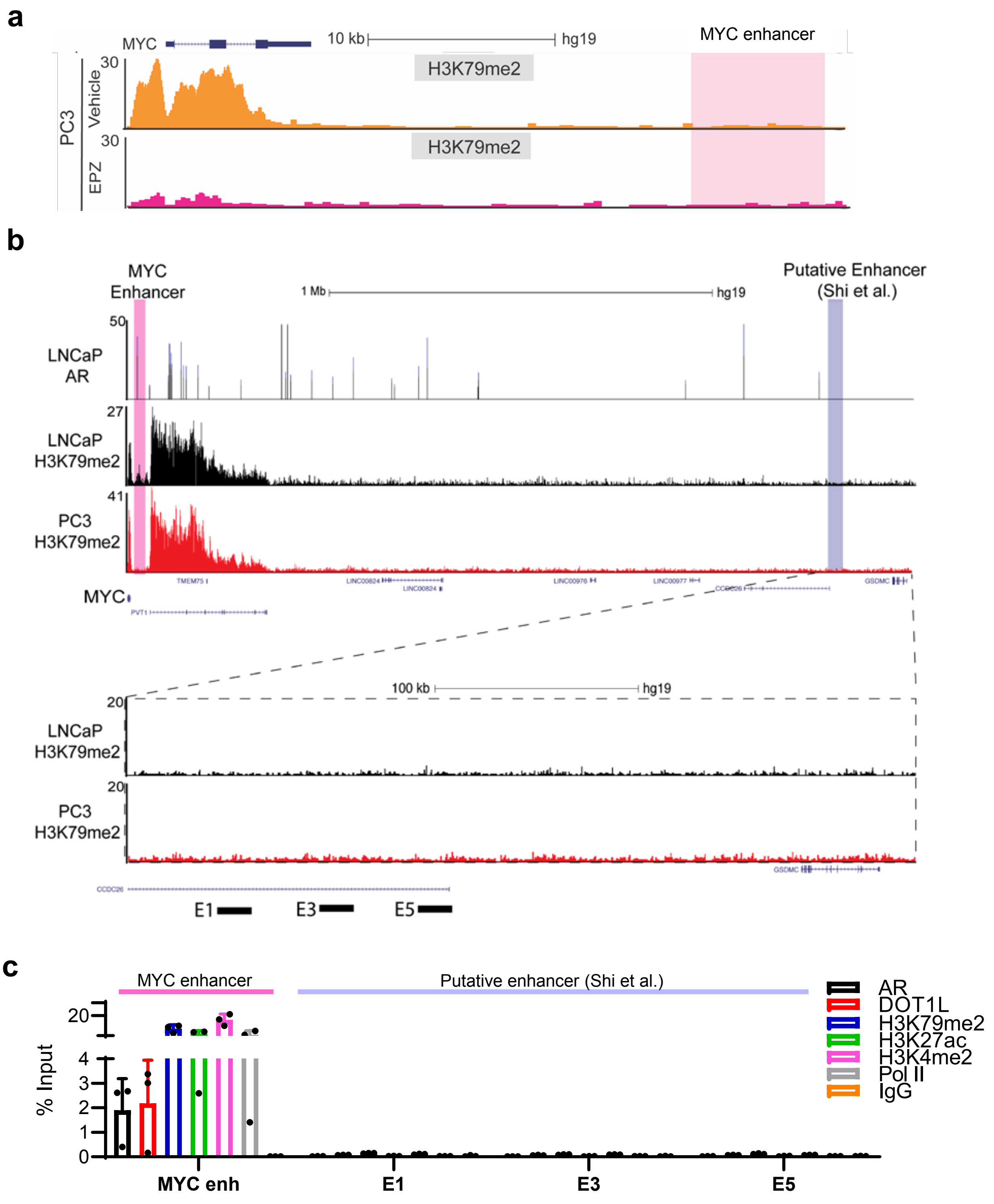

Supplementary Figure 7: (a) ChIP-seq tracks of H3K79me2 in Vehicle and EPZ treated PC3 cells (EPZ 1uM, 8 days) (b) ChIP-seq tracks (hg19) of AR (GSM353644) in LNCaP cells and H3K79me2 in Vehicle and EPZ treated PC3 cells (EPZ 1uM, 8 days) at the putative enhancer identified previously by Shi et al. (c) Enrichment of AR, DOT1L, H3K79me2, H3K27ac, H3K4me2 and RNA Pol II in LNCaP cells (vehicle treated) at the previously identified Putative enhancer at the indicated sites. The *MYC* enhancer data from Figure 7e for LNCaP DMSO is shown here for comparison. Statistical tests: P-values determined by two-tailed student's t-test n = 3 (c) independent experiments. Error bars represent s.e.m. *p<0.05, ** p<0.01, *** p<0.001, ****p<0.0001

Supplementary Fig 8.

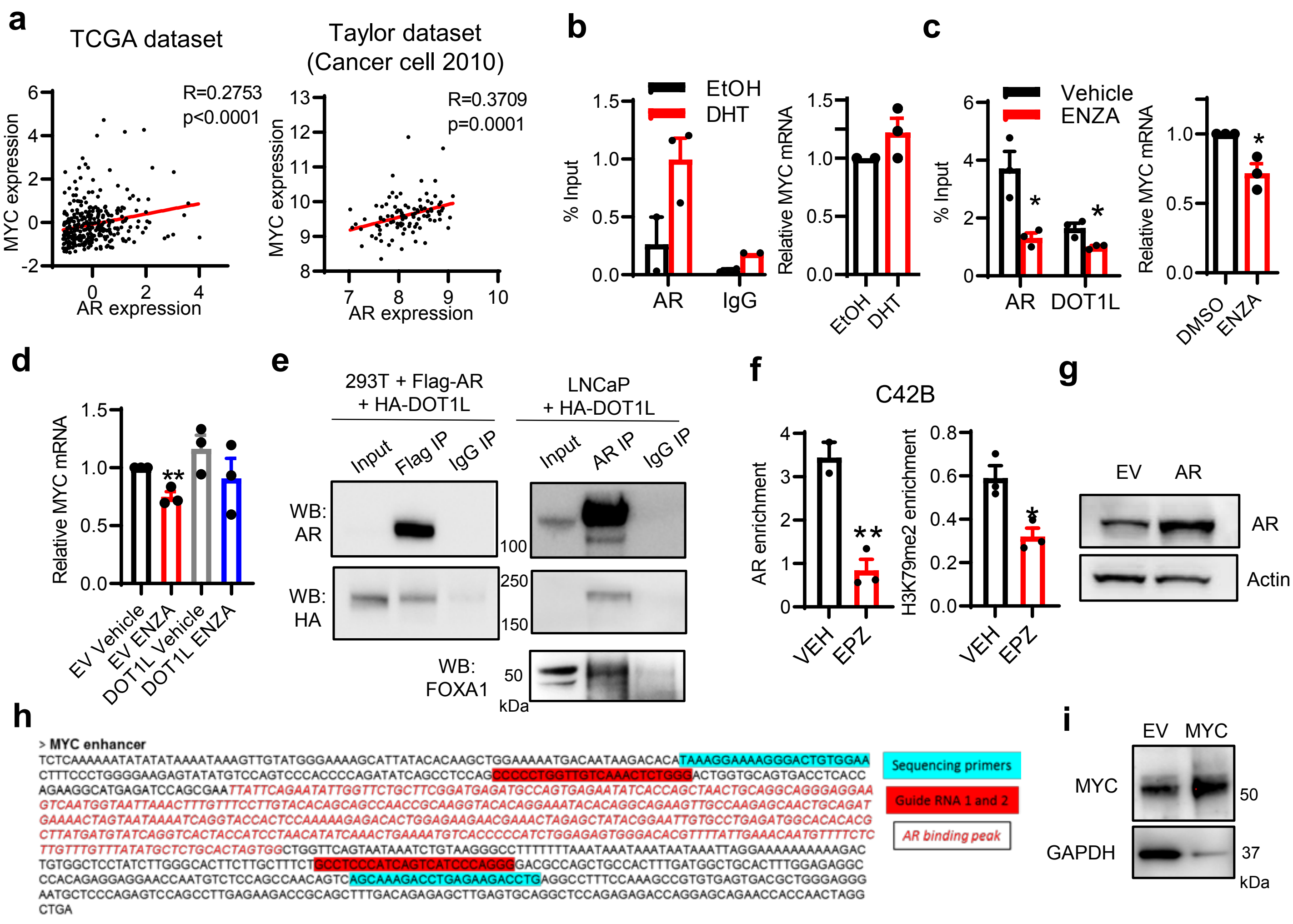

Supplementary Figure 8: (a) Correlations between gene expression of AR & MYC using data from the MSKCC dataset (n=101) and TCGA dataset (n=336) excluding patients with MYC amplifications. P-values were analyzed using Spearman's rank correlation. (b) (left) AR enrichment at *MYC* enhancer in LNCaP cells treated with Vehicle or DHT for 3 hours after 24 hours of hormone starvation in Charcoal stripped medium. (right) *MYC* expression in LNCaP cells treated with with Vehicle or DHT for 24 hours after 24 hours of hormone starvation in Charcoal stripped medium. (c) (left) AR and DOT1L enrichment at *MYC* enhancer in LNCaP cells treated with Vehicle or ENZA for 24 hours (right) *MYC* expression in LNCaP cells treated with with Vehicle or ENZA for 24 hours (d) *MYC* expression in LNCaP cells transduced with EV or DOT1L and treated with Vehicle or Enza for 24 hours. (e) (left)HA western blot performed after Flag pulldown in 293T cells expressing both Flag-AR and HA-DOT1L constructs. (right) HA and FOXA1 western blot after Flag pulldown in LNCaP cells expressing HA-DOT1L (f) (left) Enrichment of AR and H3K79me2 at the *MYC* enhancer in C42B cells treated with Vehicle or 1uM EPZ for 8 days. (g) AR protein expression in LNCaP cells transduced with EV or wild-type AR. (h) Sequence of the AR binding peak (red text) within the *MYC* enhancer. Sequencing primers are highlighted in blue and the guide RNAs in red. (i) MYC protein expression in LNCaP cells transfected with EV or MYC constructs after 2 days. Statistical tests: P-values determined by two-tailed student's t-test (a-d,f). n = 3 (a-d,f,g,i), n= 4(e) independent experiments. Error bars represent s.e.m. *p<0.05, ** p<0.01, *** p<0.001, ****p<0.0001

Supplementary Fig 9.

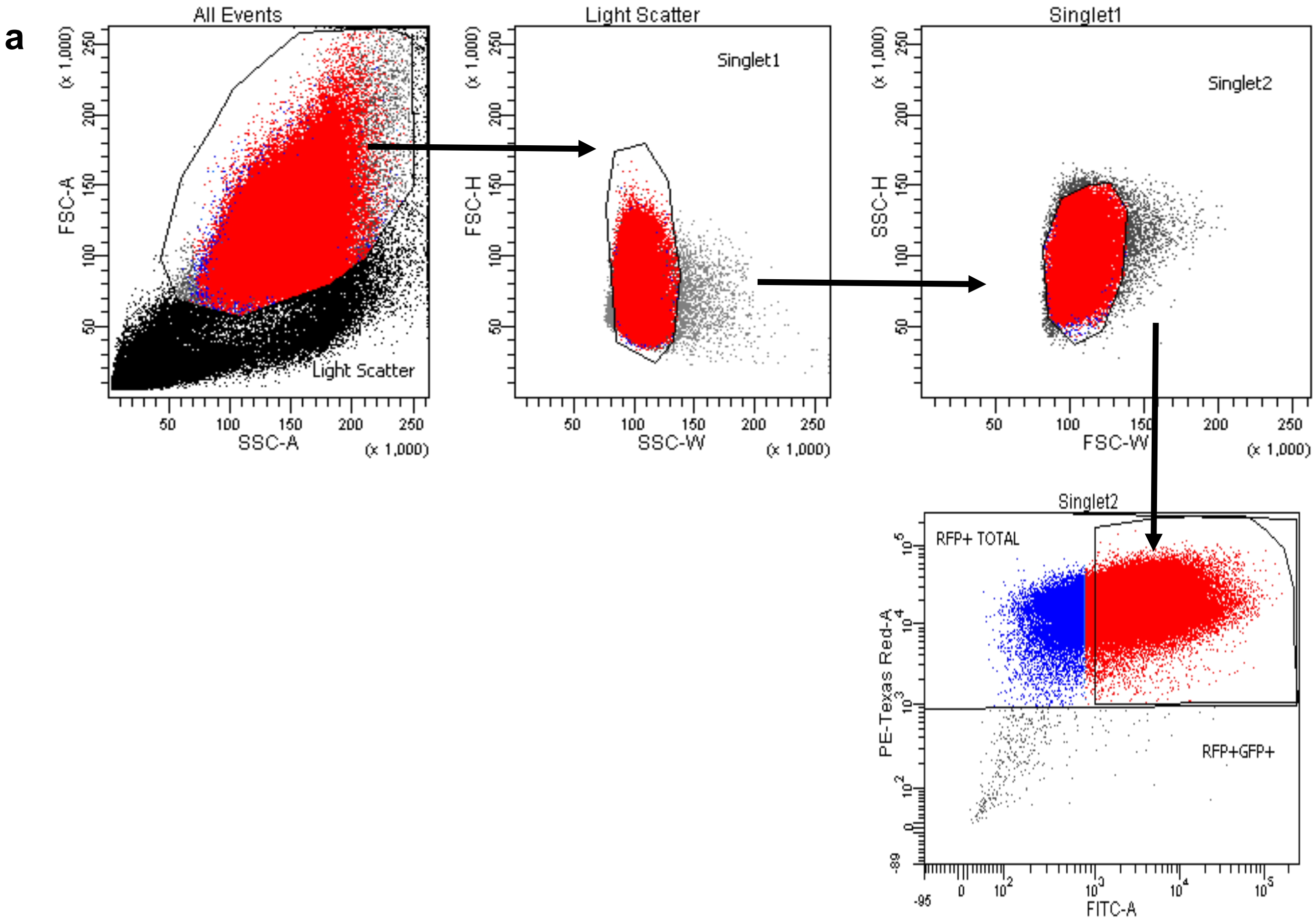

Supplementary Figure 9: (a) Gating strategy of flow cytometry analysis on LNCaP cells treated wiith Vehicle in the study described in Figure 3i. For all the analysis, the initial gating was performed on overall morphology, singlets, live cells, followed by RFP+GFP+ cells. SSC-A = Side Scatter-Area, FSC-A = Forward Scatter-Area, FSC-H = Forward Scatter-Height, FSC-W = Forward Scatter-width, SSC-W = Side Scatter-Width, SSC-H = Side Scatter-Height, FITC-A - Fluorescein isothiocyanate (FITC)-A, PE-Texas Red-A

Supplementary Table 1. qRT-PCR primers

Name	Sequence
PSA_F	TGTGTGCTGGACGCTGGA
PSA_R	CACTGCCCCATGACGTGAT
TMPRSS2_F	GGACAGTGTGCACCTCAAAGAC
TMPRSS2_R	TCCCACGAGGAAGGTCCC
AR-FL_F	TCTTGTCGTCTTCGGAAATGT
AR-FL_R	AAGCCTCTCCTTCCTCCTGTA
DOT1L_F	CAAGTTCTCGCTGCCTCACT
DOT1L_R	GTCCTGAGGGCTCAGCTTC
18SrRNA_F	GTAACCCGTTGAACCCCATT
18SrRNA_R	CCATCCAATCGGTAGTAGCG
UGT2B15_F	GTGTTGGGAATATTATGACTACAGTAAC
UGT2B15_R	GGGTATGTTAAATAGTTCAGCCAGT
UGT2B17_F	TTTTGTCGCAGGAAAAAGGAAA
UGT2B17_R	AAGCCTGAAGTGGAATGACCAA
UGT2B7_F	TTTCACAAGTACAGGAAATCATGTCAAT
UGT2B7_R	CAGCAGCTCACTACAGGGAAAAAT
HERC3_F	CTCTGGCAGATCAGCATATCATT
HERC3_R	CAGCTTTTGTATTAACCTGGGCA
MYCBP2_F	AGTCTTGGTTAGGGTATGCTCA
MYCBP2_R	GGGCTTATCCTTATGGCTGTCAT
ELL2_F	CATCACCGTACTGCATGTGAA
ELL2_R	ACTGGATTGAAGGTCGAAAAGG
NDRG1_F	CTCCTGCAAGAGTTTGATGTCC
NDRG1_R	TCATGCCGATGTCATGGTAGG
ABCC4_F	AGCTGAGAATGACGCACAGAA
ABCC4_R	ATATGGGCTGGATTACTTTGGC
TRIM49_F	GAACGAAATGTGCCATAAACCAG
TRIM49_R	TGCAGAGTAATATGCACTCGGAA
HECTD4_F	GACCGAAGACAGCCCAAAGA
HECTD4_R	AGAACATGCAGGCTCGAACA
MYC_F	TTCGGGTAGTGGAAAACCAG
MYC_R	CAGCAGCTCGAATTTCTTCC

Supplementary Table 2. ChIP qPCR primers

Name	Sequence	Purpose
PSA_F	CAGAGACCTTGATGCTTGGC	H3K79me2 ChIP
PSA_R	CCAGACTGAGGGACCCATTT	H3K79me2 ChIP
PSA_F	ACAGACCTACTCTGGAGGAAC	AR ChIP
PSA_R	AAGACAGCAACACCTTTTT	AR ChIP
TMPRSS2_F	TAGCAACACCCTCGGGTAAG	H3K79me2 ChIP
TMPRSS2_R	AAATAACCAGAGGCCGAGGT	H3K79me2 ChIP
TMPRSS2_F	TGGTCCTGGATGATAAAAAAAGTTT	AR ChIP
TMPRSS2_R	ACATACGCCCCACAACAGA	AR ChIP
UGT2B15_F	TCATGACCCCTCTGAACAAGC	AR ChIP
UGT2B15_R	CTCTGGAAGCTGTGGAAAGGT	AR ChIP
UGT2B17_F	TGAGCTGCATCAGCAGAAAGA	AR ChIP
UGT2B17_R	AAGCACTGCATAAGACCAGGA	AR ChIP
HERC3_F	GGGGACCAAGAAACACCTTT	AR ChIP
HERC3_R	GGAGGGAAAAGCACTGACTG	AR ChIP
ELL2_F	CCCATTCAGAACAGAAAGTCC	AR ChIP
ELL2_R	TTTGCTTGCAGTTACCCAAA	AR ChIP
ABCC4_F	TAGCTCTGCACGAAACTGGA	AR ChIP
ABCC4_R	TTGAGTCCCGTCTGTTTTCC	AR ChIP
MYC_F	GCAGGGAGGAAGTCAATGGT	Enhancer
MYC_R	TCATCTGCAGTTGCTCTTGG	Enhancer
FKBP5_F	ACCCTTCAGTGTGGTTCAGG	H3K79me2 ChIP
FKBP5_R	ACCACGAGCTCAAACTGCTT	H3K79me2 ChIP
NKX3.1_F	GATGGGTGGGAGGAGATGA	H3K79me2 ChIP
NKX3.1_R	TGTCTTGGACAAGCGGAG	H3K79me2 ChIP
E1_F	AGGAGCCCACCTTCTCATTT	Putative enhancer
E1_R	ACATTGCAAGAGTGGCTGTG	Putative enhancer
E3_F	TGGCAGTGGTCACAGTTCTC	Putative enhancer
E3_R	CTCTGCACCTTGAGCATTGA	Putative enhancer
E5_F	CAATACTTTCCGGCCATTTC	Putative enhancer
E5_R	GACGTTGGCCACTTCATCTT	Putative enhancer
Negative control_F	GGTCAGGCCAACTTGATTGT	Negative control
Negative control_R	AATTTGTGTTGGGCCACATT	Negative control
HECTD4_F	GTCCGAGTCGCCATACCC	MYC ChIP
HECTD4_R	CAACATGGCGTCTCACTGAC	MYC ChIP
MYCBP2_F	CTCCTCGCACATGCTCAGTA	MYC ChIP
MYCBP2_R	ACTTCAGATTCCGCACAACC	MYC ChIP
CDC25A_F	GTGAAGGCGCTATTTGGCG	MYC ChIP
CDC25A_R	TGGTTGCTCATAATCACTGCC	MYC ChIP
MYB_F	CCAACTGTTCACGCAGACCT	MYC ChIP
MYB_R	CTTCTGATGCTGGTGCCATT	MYC ChIP
