# Supplementary material for: Histone methyltransferase DOT1L coordinates AR and MYC stability in prostate cancer
Source: Nat Commun. 2020 Aug 19;11:4153. doi: 10.1038/s41467-020-18013-7 (PMC7438336; doi:10.1038/s41467-020-18013-7)
Supplement: Supplementary file 3 — Description of Additional Supplementary Files [file 41467_2020_18013_MOESM3_ESM.pdf]

Description of Additional Supplementary Files

File Name: Supplementary Data 1
Description: A list of proteins identified by Mass spectrometry performed after AR pulldown in LNCaP cells treated with Vehicle and 1uM EPZ004777 for 8 days.
