# Supplementary material for: Histone methyltransferase DOT1L coordinates AR and MYC stability in prostate cancer
Source: Nat Commun. 2020 Aug 19;11:4153. doi: 10.1038/s41467-020-18013-7 (PMC7438336; doi:10.1038/s41467-020-18013-7)
Supplement: Supplementary file 5 — Reporting Summary [file 41467_2020_18013_MOESM5_ESM.pdf]

nature research

Corresponding author(s): Dr.Sarki Abdulkadir

Last updated by author(s): Jul 8, 2020

Reporting Summary

Nature Research wishes to improve the reproducibility of the work that we publish. This form provides structure for consistency and transparency in reporting. For further information on Nature Research policies, see Authors & Referees and the Editorial Policy Checklist.

Statistics

For all statistical analyses, confirm that the following items are present in the figure legend, table legend, main text, or Methods section.

n/a	Confirmed	
☐	☒	The exact sample size (n) for each experimental group/condition, given as a discrete number and unit of measurement
☐	☒	A statement on whether measurements were taken from distinct samples or whether the same sample was measured repeatedly
☐	☒	The statistical test(s) used AND whether they are one- or two-sided<br>*Only common tests should be described solely by name; describe more complex techniques in the Methods section.*
☐	☒	A description of all covariates tested
☒	☐	A description of any assumptions or corrections, such as tests of normality and adjustment for multiple comparisons
☐	☒	A full description of the statistical parameters including central tendency (e.g. means) or other basic estimates (e.g. regression coefficient) AND variation (e.g. standard deviation) or associated estimates of uncertainty (e.g. confidence intervals)
☐	☒	For null hypothesis testing, the test statistic (e.g. F, t, r) with confidence intervals, effect sizes, degrees of freedom and P value noted<br>*Give P values as exact values whenever suitable.*
☒	☐	For Bayesian analysis, information on the choice of priors and Markov chain Monte Carlo settings
☒	☐	For hierarchical and complex designs, identification of the appropriate level for tests and full reporting of outcomes
☐	☒	Estimates of effect sizes (e.g. Cohen's d, Pearson's r), indicating how they were calculated

Our web collection on statistics for biologists contains articles on many of the points above.

Software and code

Policy information about availability of computer code

Data collection	ChIP-seq data was collected by Basespace (Illumina). qPCR data was collected using QuantStudio Software v1.3 (for QuantStudio 6 Flex Real-Time PCR Systems), BD FACSDiva 8.0.1 for Flow analysis.
Data analysis	GraphPad Prism 8 was used for all analyses unless stated otherwise. Raw data (CEL files) were processed and normalized using Bioconductor oligo package. Differentially expressed genes were identified by the Bioconductor limma package. Gene set enrichment analysis (GSEA) was done using C2 curated and C6 oncogenic gene sets. For ChIP-seq, Sequence reads were aligned using Burrows-Wheeler Alignment (BWA) Tool Version 0.6.1. Peak identification, overlapping, subtraction and feature annotation of enriched regions were performed using Hypergeometric Optimization of Motif EnRichment suite (HOMER v4). Weighted venn diagrams were created by R package Vennerable. Differentially enriched genes were loaded into Enrichr website for ChIP Enrichment analysis (ChEA).

For manuscripts utilizing custom algorithms or software that are central to the research but not yet described in published literature, software must be made available to editors/reviewers. We strongly encourage code deposition in a community repository (e.g. GitHub). See the Nature Research guidelines for submitting code & software for further information.

Data

Policy information about availability of data

All manuscripts must include a data availability statement. This statement should provide the following information, where applicable:

- Accession codes, unique identifiers, or web links for publicly available datasets
- A list of figures that have associated raw data
- A description of any restrictions on data availability

The data that support the findings of this study (Microarray and ChIP-seq) are available in the GEO under accession GSE135575 (https://www.ncbi.nlm.nih.gov/geo/query/acc.cgi?acc=GSE 135575).ChIP-seq data (Fig 2l, Fig 3n, Fig 7a) is deposited in GSE135574 and Microarray data (Fig 2j, Fig 3b, Fig 5a) is deposited in GSE135573. The expression and ChIP-seq data referenced in this study is available from the NCBI GEO website. The ChIP-seq tracks of AR in LNCaP cells treated with R1881 (GSM353644) was downloaded from GSE14092. H3K27ac in LNCaP cells (GSM686937) is from GSE27823. ChIP-seq tracks of AR and H3K27ac in 3 patient

samples (223T, 227T, 229T) was extracted from GSE120738. MYC ChIP-seq track was downloaded from GSE73994. Gene expression data were downloaded from the NCBI Geo from GSE35988, GSE6919, GSE3325, GSE21032, GSE94767 GSE70768, GSE40272, GSE74367, GSE3971, GSE20842, GSE7696, GSE9348, GSE13159, and GSE13507. Source data underlying figures provided as Source data file. All the other data supporting the findings of this study are available within the article and its supplementary information files and from the corresponding author upon reasonable request.

Field-specific reporting

Please select the one below that is the best fit for your research. If you are not sure, read the appropriate sections before making your selection.

☒ Life sciences ☐ Behavioural & social sciences ☐ Ecological, evolutionary & environmental sciences

For a reference copy of the document with all sections, see nature.com/documents/nr-reporting-summary-flat.pdf

Life sciences study design

All studies must disclose on these points even when the disclosure is negative.

Sample size	No study size calculation was performed for in vitro experiments. Sample size was chosen based on our prior studies using the same types of assays, as well as published literature, to ensure statistically significant results. For in vitro studies used a standard n=3. For the in vivo experiment, n=5 which is enough to provide 90% power to detect a difference of approximately 2 SD between the groups with type I error rate of 5%.
Data exclusions	No data was excluded from this analysis.
Replication	n=3 biological replicates for cell assays, western blots, Microarray, qRT-PCR, ChIP-qPCR in Figures 2,3,4,5, 6 and 7; All replication attempts were successful. n=1 biological replicate ChIP-seq
Randomization	Cells treated with Vehicle or EPZ004777 and injected into randomized mice of similar age (n=5 per group). Randomization was not relevant for other experiments, as they were performed in cell lines.
Blinding	Investigators were not blinded to the animal treatment groups as the same investigator both planned and performed the experiment. For the IHC, both staining and analysis was completed in a blinded manner.

Reporting for specific materials, systems and methods

We require information from authors about some types of materials, experimental systems and methods used in many studies. Here, indicate whether each material, system or method listed is relevant to your study. If you are not sure if a list item applies to your research, read the appropriate section before selecting a response.

Materials & experimental systems

n/a	Involved in the study	
☐	☒	Antibodies
☐	☒	Eukaryotic cell lines
☒	☐	Palaeontology
☐	☒	Animals and other organisms
☐	☒	Human research participants
☒	☐	Clinical data

Methods

n/a	Involved in the study	
☐	☒	ChIP-seq
☐	☒	Flow cytometry
☒	☐	MRI-based neuroimaging

Antibodies

Antibodies used	H3K79me2 (ab177184; Abcam, 1: 1000), AR (RB-9030-P1; Thermo Fischer, 1: 1000), PSA (A0562; Dako, 1: 1000), Actin (sc-1616; Santa Cruz Biotech, 1: 1000), Histone H3 (ab1791; Abcam, 1: 3000), GAPDH (sc-20357; Santa Cruz Biotech, 1: 1000), DOT1L (EMD Millipore MABE425, Abcam ab72454, 1: 100), MYC (Abcam ab32072, 1: 1000), Halotag (Promega G9211, 1: 1000), and Ubiquitin (Cell Signaling Technology 3936, 1: 1000) c-Myc (phospho S62, Abcam ab51156, 1: 1000), Anti-c-Myc (phospho T58, Abcam ab185655, 1: 1000), Flag (Sigma-Aldrich F1804, 1: 1000), Myc-Tag (Cell Signaling Technology 2276S, 1: 1000), FOXA1 (Abcam ab5089, 1: 500)
Validation	Validation was carried out by the manufacturer.<br>CST - "At Cell Signaling Technology (CST), we understand that there is no single assay that can determine the validity of an antibody. Confirming that an immunoreagent is sufficiently specific and sensitive depends on the application and protocol being used, the type and quality of sample being analyzed, and the inherent biophysical properties of the antibody itself.<br><br>To ensure our antibodies will work in your experiment, we adhere to the Hallmarks of Antibody Validation™, six complementary strategies that can be used to determine the functionality, specificity, and sensitivity of an antibody in any given assay. CST adapted the work by Uhlen, et. al., (“A Proposal for Validation of Antibodies.” Nature Methods (2016)) to build the Hallmarks of

Antibody Validation, based on our decades of experience as an antibody manufacturer and our dedication to reproducible science."

Abcam - "Antibodies are validated in western blot using lysates from cells or tissues that we have identified to express the protein of interest. Once we have determined the right lysates to use, western blots are run and the band size is checked for the expected molecular weight. We will always run several controls in the same western blot experiment, including positive lysate and negative lysate. When possible, we also include knock-out (KO) cell lines as a true negative control for our western blots. We are always increasing the number of KO-validated antibodies we provide. In addition, we run old stock alongside our new stock. If we know the old stock works well, this also acts as a suitable positive control.If the western blot result gives a clear clean band and we are happy with the result from the control lanes, these antibodies will be passed and added to the catalog."

Eukaryotic cell lines

Policy information about cell lines

Cell line source(s)	ATCC (22rv1, LNCaP, RWPE-1, VCaP, 293T). PC3, DU145, C42B were a gift from Dr. D. Chakravarti.
Authentication	PC3, DU145, C42B were authenticated by ATCC using STR profiling.
Mycoplasma contamination	All cells were tested negative for mycoplasma contamination.
Commonly misidentified lines (See ICLAC register)	None.

Animals and other organisms

Policy information about studies involving animals; ARRIVE guidelines recommended for reporting animal research

Laboratory animals	Male Mice, NOD-SCID strain (for cell lines) and NSG strain (for PDX) male, 6-8 weeks old
Wild animals	No wild animals were used.
Field-collected samples	No field collections were done.
Ethics oversight	All experiments and procedures were performed in compliance with ethical regulations and the approval of the Northwestern University Institutional Animal Care and Use Committee (IACUC).

Note that full information on the approval of the study protocol must also be provided in the manuscript.

Human research participants

Policy information about studies involving human research participants

Population characteristics	Archived RNA samples obtained from metastases of patients with treatment resistant metastatic prostate cancer.
Recruitment	Exempt study. Archived de-identified samples were used.
Ethics oversight	Archived de-identified samples were used. Originally, informed consent was obtained from all patients under the oversight of Institutional Review Board (IRB) of University of Washington.

Note that full information on the approval of the study protocol must also be provided in the manuscript.

ChIP-seq

Data deposition

☒ Confirm that both raw and final processed data have been deposited in a public database such as GEO.

☒ Confirm that you have deposited or provided access to graph files (e.g. BED files) for the called peaks.

Data access links<br>*May remain private before publication.*	https://genome.ucsc.edu/s/vrajita/Prostate_H3K79me2<br>Data available in GEO.(GSE135575)
Files in database submission	ChIP-seq BED tracks:<br>PC3_EPZ_INPUT<br>PC3_EPZ_H3K79ME2<br>PC3_DMSO_INPUT<br>PC3_DMSO_H3K79ME2<br>LNCAP_EPZ_INPUT<br>LNCAP_EPZ_H3K79ME2

LNCAP_DMSO_INPUT
LNCAP_DMSO_H3K79ME2

Genome browser session (e.g. UCSC): https://genome.ucsc.edu/s/vrajita/Prostate_H3K79me2

Methodology

Replicates: n=1

Sequencing depth:

sample name	reads	mapped reads	map %
PC3_EPZ_INPUT	31,076,940	28,808,826	93%
PC3_EPZ_H3K79ME2	24,748,002	23,567,245	95%
PC3_DMSO_INPUT	27,869,621	26,056,828	93%
PC3_DMSO_H3K79ME2	21,834,547	21,000,340	96%
LNCAP_EPZ_INPUT	31,646,933	29,504,918	93%
LNCAP_EPZ_H3K79ME2	30,375,643	26,726,436	88%
LNCAP_DMSO_INPUT	24,069,215	22,574,011	94%
LNCAP_DMSO_H3K79ME2	20,951,630	20,110,602	96%

Antibodies: H3K79me2 (Abcam; ab177184)

Peak calling parameters: Peak identification, overlapping, subtraction and feature annotation of enriched regions were performed using Hypergeometric Optimization of Motif EnRichment suite (HOMER).

Data quality: 10% FDR (default) was used for broad peak calling.

Software: Differentially enriched genes were loaded into Enrichr website for ChIP Enrichment analysis (ChEA)

Flow Cytometry

Plots

Confirm that:

☒ The axis labels state the marker and fluorochrome used (e.g. CD4-FITC).

☒ The axis scales are clearly visible. Include numbers along axes only for bottom left plot of group (a 'group' is an analysis of identical markers).

☒ All plots are contour plots with outliers or pseudocolor plots.

☒ A numerical value for number of cells or percentage (with statistics) is provided.

Methodology

Sample preparation: LNCaP cells with GFP-ARE construct were treated with or without EPZ004777 for 8 days. On Day 8, cells were trypsinized and dissociated and resuspended in Phenol-free RPMI (1X10^6 cells/ml)

Instrument: BD FACSAria

Software: BD FACSDiva 8.0.1

Cell population abundance: No sorting was performed, only analysis of the sample.

Gating strategy: Initial gating was performed on overall morphology, singlets and live cells. Cells were then gated for RFP+ (99%,Texas Red-A) followed by measurement of GFP+ population (FITC). The gating strategy is provided in Supplementary Figure 9.

☒ Tick this box to confirm that a figure exemplifying the gating strategy is provided in the Supplementary Information.
